# Whole Genome Sequencing of *Mycobacterium tuberculosis* under routine conditions in a high-burden area of multidrug-resistant tuberculosis in Peru

**DOI:** 10.1371/journal.pone.0304130

**Published:** 2024-06-11

**Authors:** Zully M. Puyén, David Santos-Lázaro, Aiko N. Vigo, Vidia V. Cotrina, Nathaly Ruiz-Nizama, Miriam J. Alarcón, Belisa Asto, Teresa Huamán, David A. J. Moore

**Affiliations:** 1 Laboratorio de Referencia Nacional de Micobacterias, Instituto Nacional de Salud, Lima, Perú; 2 Escuela de Medicina, Universidad Privada de Ciencias Aplicadas, Lima, Perú; 3 Universidad Peruana Cayetano Heredia, Lima, Perú; 4 London School of Hygiene & Tropical Medicine, London, United Kingdom; International Centre for Diarrhoeal Disease Research, BANGLADESH

## Abstract

Whole Genome Sequencing (WGS) is a promising tool in the global fight against tuberculosis (TB). The aim of this study was to evaluate the use of WGS in routine conditions for detection of drug resistance markers and transmission clusters in a multidrug-resistant TB *hot-spot* area in Peru. For this, 140 drug-resistant *Mycobacterium tuberculosis* strains from Lima and Callao were prospectively selected and processed through routine (GenoType MTBDR*sl* and BACTEC MGIT) and WGS workflows, simultaneously. Resistance was determined in accordance with the World Health Organization mutation catalogue. Agreements between WGS and BACTEC results were calculated for rifampicin, isoniazid, pyrazinamide, moxifloxacin, levofloxacin, amikacin and capreomycin. Transmission clusters were determined using different cut-off values of Single Nucleotide Polymorphism differences. 100% (140/140) of strains had valid WGS results for 13 anti-TB drugs. However, the availability of final, definitive phenotypic BACTEC MGIT results varied by drug with 10–17% of invalid results for the seven compared drugs. The median time to obtain results of WGS for the complete set of drugs was 11.5 days, compared to 28.6–52.6 days for the routine workflow. Overall categorical agreement by WGS and BACTEC MGIT for the compared drugs was 96.5%. Kappa index was good (0.65≤k≤1.00), except for moxifloxacin, but the sensitivity and specificity values were high for all cases. 97.9% (137/140) of strains were characterized with only one sublineage (134 belonging to “lineage 4” and 3 to “lineage 2”), and 2.1% (3/140) were mixed strains presenting two different sublineages. Clustering rates of 3.6% (5/140), 17.9% (25/140) and 22.1% (31/140) were obtained for 5, 10 and 12 SNP cut-off values, respectively. In conclusion, routine WGS has a high diagnostic accuracy to detect resistance against key current anti-TB drugs, allowing results to be obtained through a single analysis and helping to cut quickly the chain of transmission of drug-resistant TB in Peru.

## Introduction

Tuberculosis (TB) is an infectious disease caused by *Mycobacterium tuberculosis* (MTB). Until the COVID-19 pandemic, TB was the leading cause of death from a single infectious agent worldwide, above HIV/AIDS and malaria, with 10 million new cases and an estimated 1.5 million deaths in 2020 [1]. Peru is considered one of the 30 countries in the world with the highest burden of multidrug-resistant TB (MDR-TB) and the country with the highest MDR-TB burden in the Latin American region [2]. MDR-TB is distributed throughout the country, but disproportionately concentrated in the capital city of Lima and Callao which account for 78% of all cases [3].

Since 2018, the World Health Organization (WHO) has endorsed the use of next generation sequencing technologies, including Whole Genome Sequencing (WGS) for the detection of mutations associated with MTB resistance [4]. WGS allows the simultaneous interrogation of multiple regions of the MTB genome where well-characterized resistance-conferring mutations may be detected, facilitating drug susceptibility testing (DST) of multiple agents at the same time without additional effort or cost. This improves diagnostic accuracy in comparison to previous molecular methodologies targeted on a limited number of genetic regions. In 2021, the WHO has curated a catalogue of high-confidence mutations that confer resistance to 13 anti-TB drugs, allowing a uniform interpretation of the WGS results in the genotypic determination of resistance [5]. In addition, the use of the WGS has opened the door to a new genomic epidemiology of TB, and we can now observe transmission with unprecedented resolution.

The Peruvian National Institute for Health (Instituto Nacional de Salud–INS) has undertaken WGS pilot studies in recent years supported by the Peruvian government national science council (CONCYTEC) and the Newton Fund of the UK Government, which have demonstrated the feasibility of WGS implementation in the public health sector. This present work sought to extend that experience to establish and evaluate the use of WGS in a routine workflow and describe the profile of drug resistance conferring mutations and the degree to which MDR-TB strains were genomically clustered in our setting. As countries start to consider implementation of WGS into their TB DST workflows we wish to share our experience from a high MDR-TB burden country with nationally centralized second-line DST.

## Materials and methods

### Design and study population

A prospective study was carried out at the National Reference Laboratory for Mycobacteria (Laboratorio de Referencia Nacional de Micobacterias ‐ LRNM) of the INS in collaboration with the London School of Hygiene and Tropical Medicine (LSHTM). We selected positive MTB cultures that were previously isolated from clinical samples of patients with pulmonary or extrapulmonary TB and that were sent to the LRNM for routine DST in a total time of 45 days (from 12/1/2021 to 12/30/2021 and from 03/10/2022 to 03/24/2022). Drug-resistant MTB strains were selected daily according to the inclusion criteria: i) positive solid culture (Lowenstein-Jensen) for MTB with at least 10 colonies, ii) positive solid culture with previous phenotypic or genotypic result demonstrating rifampicin and/or isoniazid resistance (the key national criterion for sending strains from regional laboratories to LRNM for DST), and iii) positive solid culture from districts of the provinces of Lima and Callao, which are considered the main MDR-TB *hot-spot* areas in Peru.

### Ethics statement

The study was reviewed and approved by the Institutional Research Ethics Committee of the INS (OC-019-21). All personal information was anonymized using a dual coding system specific to this study. The procedures of the present study did not interfere with the established laboratory workflow for routine TB diagnosis and DST. Likewise, the WGS results did not influence the clinical treatment of the patients since they are currently not yet considered for use in clinical care in Peru. Informed consent was not required.

### Conventional laboratory diagnostic processes

Cultures were routinely processed for DST through genotypic and phenotypic methodologies according to the existing laboratory workflow. Samples were first evaluated by genotypic DST using Line Probe Assay (LPA) GenoType MTBDR*sl* v2 kit (Hain Lifescience GmbH Germany) for the detection of resistance to fluoroquinolones and second-line injectables drugs. After that, the same culture was sent for phenotypic DST using the liquid culture system BACTEC MGIT 960 (Becton Dickinson®, Sparks, MD, USA) for evaluation of resistance to first- (pyrazinamide [100 μg/ml]) and second-line drugs (amikacin [1.0 μg/ml], capreomycin [2.5 μg/ml], moxifloxacin [1.0 μg/ml] and levofloxacin [1.0 μg/ml]). In a limited number of cases, resistance tests against isoniazid [0.1 μg/ml] and rifampicin [0.5 or 1.0 μg/ml] were also performed as required. According to the established laboratory workflow, results with phenotypic or genotypic resistance to any second-line drugs were subsequently evaluated for susceptibility to new (bedaquiline [1.0 μg/ml], delamanid [0.06 μg/ml]) and repurposed drugs (clofazimine [1.0 μg/ml], linezolid [1.0 μg/ml]) (S1 Fig). Critical concentrations were set according to recommended by the WHO at the date of testing [6, 7].

### DNA extraction and Whole Genome Sequencing

Genomic DNA extraction was performed from the original solid culture sent to the LRNM. Culture slants were obtained at the same moment of sample extraction for routine LPA and processed immediately in order to avoid excessive manipulations in the culture tube and minimise risk of contamination. MTB colonies were pre-incubated with lysozyme buffer for two hours. Subsequent DNA extraction was performed using the GeneJET Genomic DNA Purification kit (Thermo Fisher Scientific, Waltham, USA) according to manufacturer’s recommendations for gram-positive specimens. DNA concentration was quantified using the Qubit dsDNA HS Assay kit (Thermo Fisher Scientific, Waltham, USA). Sequencing libraries were prepared using Nextera XT Library Preparation kit generating paired-end sequencing reads. Whole genome sequencing was carried out at INS using Illumina MiSeq platform (Illumina, San Diego, USA) with 600 cycles v3 cartridges. Raw sequencing data was submitted to the National Center for Biotechnology Information (NCBI) Sequence Read Archive (SRA) under BioProject ID: PRJNA906938.

### Bioinformatics analysis

Sequencing data was analysed on a bioinformatic server of the LRNM and were entirely set on the Ubuntu distribution of Linux using an *in-house* pipeline that automated all processes. The quality of the sequencing reads was initially evaluated using the program FastQC v0.11 [8]. Paired-end reads were filtered with Trimmomatic v0.39 (*headcrop*:*10*, *leading*:*10*, *trailing*:*10*, *slidingwindow*:*4*:*20*, *minlen*:*40*) [9]. The purity of the sequenced sample was evaluated using Kraken2 v2.0.7 [10]. Filtered reads were mapped against the H37Rv reference genome (NC_000962.3) using BWA v0.7.17 [11]. Lineage and resistant variants were obtained using the command-line version of TB-profiler v4.1.1 [12] taking as input the bam file generated in previous mapping process. The software was set to obtain resistant variants with a minimum depth of 10x and a minimum allele frequency of 5%. The mutation database v’21c072d’ of TBDB repository (https://github.com/jodyphelan/tbdb/tree/who) was used, which includes only WHO catalogue mutations (‘associated with resistance’ and ‘associated with resistance–interim’ variants) for 13 WHO-endorsed anti-TB drugs (isoniazid, rifampicin, ethambutol, pyrazinamide, moxifloxacin, levofloxacin, ethionamide, streptomycin, kanamycin, amikacin, capreomycin, delamanid, linezolid) [5]. Genome-wide variant calling was performed using GATK v4.1.8.1 (*—min-base-quality-score 20*,*—stand-call-conf 30*,*—sample-ploidy 2*) [13]. High-confident calls were obtained using a hard-filtering approach to select variants with a minimum mapping quality of 60, a minimum depth of 10x and a minimum percentage of reads supporting alternate allele of 75%. Individual *fasta* files were generated using genome-wide high confident Single Nucleotide Polymorphisms (SNP). Genomic coordinates with missing genotypes in a minimum of 10% of all strains and variants located in repetitive regions (PE, PPE and PE-PGRS genes) or in resistant-associated genes were excluded. Transmission clusters were determined using well established cut-off values of 5, 10 and 12 SNP pairwise distance [14–17]. SNP distances were calculated using the R package Ape v5.4 [18]. Maximum parsimony tree was generated using phangorn v2.10 [19] package from R v4.2.1.

### Statistical analysis

Agreements between WGS and routine methods were determined for rifampicin, isoniazid, pyrazinamide, moxifloxacin, levofloxacin, amikacin and capreomycin. Categorical agreement, sensitivity (or positive agreement) and specificity (or negative agreement) were calculated. Categorical agreement was defined as the proportion of concordant susceptibility or resistance results between WGS and routine methods over the total number of tests. Sensitivity was defined as the proportion of concordant resistant results between WGS and BACTEC MGIT over the total number of resistant results by BACTEC MGIT. Specificity was defined as the proportion of concordant susceptible results between WGS and BACTEC MGIT over the total number of susceptible results by BACTEC MGIT. Similarly, positive and negative agreements were calculated taking the LPA GenoType MTBDR*sl* as a comparator method instead of the gold standard BACTEC MGIT. For statistical agreement, the Cohen’s Kappa coefficient (k) was performed in the program R v4.2.1 using the package vcd v1.4 (https://cran.r-project.org/web/packages/vcd). The strength of agreement was established as Slight (0.00≤k≤0.20), Fair (0.21<k≤0.40), Moderate (0.41<k≤0.60), Substantial (0.61<k≤0.80) or Almost perfect (0.81<k≤1.00) as previously proposed [20].

In-depth analysis of discordant results between WGS and BACTEC MGIT was performed. Finally, the laboratory turnaround times taken for each part of the process were measured and compared between WGS and routine workflow taking as the starting point the moment when the solid culture arrives from a referring laboratory at the LRNM for DST and the temporal censoring point as the time when the validated results report was generated (after carrying out the additional processing runs for repetitions, if necessary).

## Results

### Demographic and genetic characteristics of samples

A total of 140 strains were included in this analysis of which 74.3% were isolated from men. 84.3% (118/140) of strains were isolated in Lima, most (n = 43) from the Lima-Centro area, and 15.7% (22/140) from Callao. In detail, the strains formed a representative group since they came from the districts with the highest burden of drug-resistant TB in Peru (S2 Fig). 61.4% of strains achieved 3+ growth and 97.9% (137/140) had an eugonic appearance (Table 1). The number of strains for which valid results were obtained differed between methodologies. By WGS a complete set of valid susceptibility results was obtained for all 13 anti-TB drugs for all 140 strains. However, for BACTEC MGIT the number of valid results differed by drug: 90% (17/19) for isoniazid, 83% (47/57) for rifampicin, 84% (116/139) for pyrazinamide, 86% (120/139) for moxifloxacin, levofloxacin, amikacin and capreomycin, 100% (14/14) for bedaquiline, clofazimine and linezolid, and 93% (13/14) for delamanid. By WGS 64 MDR-TB, eight Pre extensively drug-resistant TB (Pre-XDR-TB), 47 isoniazid-monoresistant TB, six rifampicin-monoresistant TB, two with additional resistance and 13 sensible strains were identified.

**Table 1:** Epidemiological characteristics of the strains included in the study.

Characteristics	N	%
**Sex**		
Male	104	74.3
Female	36	25.7
**Age**		
< 15 years	1	0.7
15–34 years	69	49.3
35–54 years	41	29.3
> 54 years	28	20.0
Unknown	1	0.7
Median (IQR)	34 (25–49)	
**Location**		
Lima-Provincias	5	3.6
Lima-Norte	18	12.9
Lima-Centro	43	30.7
Lima-Este	28	20.0
Lima-Sur	24	17.1
Callao	22	15.7
**Growth**		
+	9	6.4
++	45	32.1
+++	86	61.4
**Morphology**		
Eugonic	137	97.9
Dysgonic	3	2.1

**IQR**: Interquartile range

### Time to obtain results

**Fig 1:**
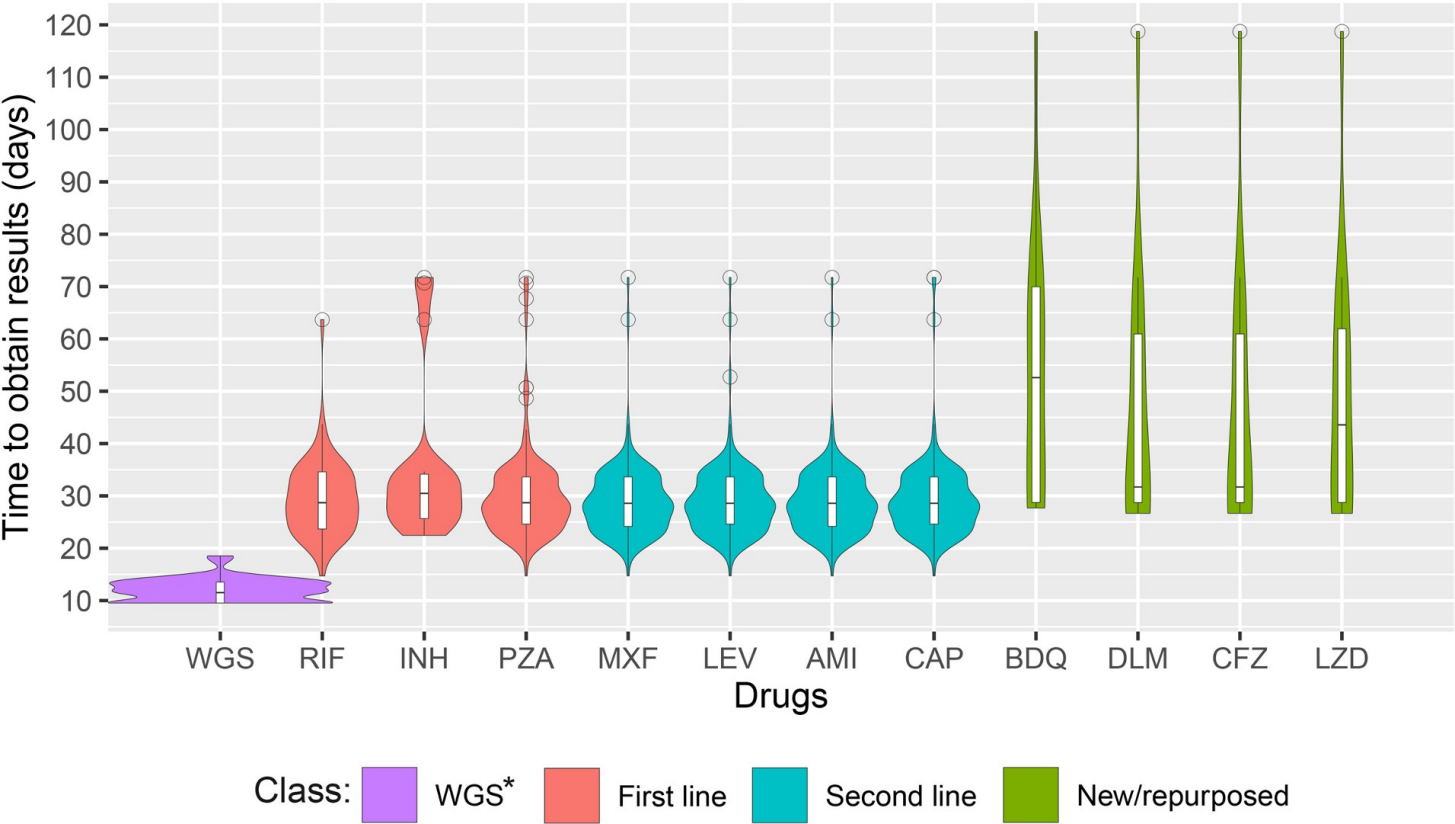
Comparison of time to obtain final results between WGS and routine workflows. Violin plots show distribution of times taken to obtain sample results for each drug analysed according to LRNM´s routine workflow. Median and interquartile range is plotted using internal boxplots. RIF: rifampicin, INH: isoniazid, PZA: pyrazinamide, MXF: moxifloxacin, LEV: levofloxacin, AMI: amikacin, CAP: capreomycin, BDQ: bedaquiline, DLM: delamanid, CFZ: clofazimine, LZD: linezolid. * The WGS results cover the times to results for 13 anti-TB drugs.

Median time to final result availability for WGS was 11.5 days (range 9.5–18.5). For routine workflow median time to final result availability varied by drug but was 30.5 days for isoniazid and around 29 days for all of rifampicin, pyrazinamide, moxifloxacin, levofloxacin, amikacin and capreomycin. For new and repurposed drugs, for which processing often followed initial BACTEC DST, median times to result availability were 52.6 days for bedaquiline, 43.6 for linezolid, and 31.7 days for clofazimine and delamanid (Fig 1). The overall median delay to availability of a complete set of valid phenotypic DST results for first- and second-line drugs was 29 days (range 14.7–71.7) rising to 40 days (range 27.7–118.7) for those additionally requiring DST for new and repurposed drugs (S1 Table). A second processing run on BACTEC MGIT was required for 18.6% (26/140) samples either because of culture contamination, lack of culture growth or need for additional evaluation of new drugs. Three samples required a third stage of DST processing. However, despite additional processing runs, it was not possible to obtain a complete set of valid results for 13.6% (19/140) of samples.

### Diagnostic accuracy

Overall categorical agreement between BACTEC MGIT and WGS for the seven compared drugs was 96.5%, ranging between 93.3–100%. According to kappa index isoniazid, pyrazinamide, levofloxacin, amikacin and capreomycin exhibited an almost perfect agreement, whereas rifampicin had a substantial agreement and moxifloxacin a fair agreement. All compared drugs showed high values of sensitivity and specificity, including drugs that presented lower kappa values (Table 2). Delamanid (13 strains) and linezolid (14 strains) had categorical agreement of 100%.

**Table 2:** Precision diagnostic results of WGS routine diagnosis.

Drug	WGS	BACTEC		CA (%)	Sensitivity	Specificity	Kappa Value (CI)	SE
		R	S					
**Rifampicin**	R	2	2	95.7	1.00	0.96	0.65 (0.20–1.00)	0.23
	S	0	43					
**Isoniazid**	R	7	0	94.1	0.88	1.00	0.88 (0.66–1.00)	0.12
	S	1	9					
**Pyrazinamide**	R	44	2	94.8	0.92	0.97	0.89 (0.81–0.98)	0.04
	S	4	66					
**Moxifloxacin**	R	2	8	93.3	1.00	0.93	0.31 (-0.02–0.65)	0.17
	S	0	110					
**Levofloxacin**	R	9	1	98.3	0.90	0.99	0.89 (0.74–1.00)	0.08
	S	1	109					
**Amikacin**	R	2	0	100.0	1.00	1.00	1.00 (1.00–1.00)	0.00
	S	0	118					
**Capreomycin**	R	8	0	99.2	0.89	1.00	0.94 (0.81–1.00)	0.06
	S	1	111					

**WGS**: Whole genome sequencing, **CA**: Categorical agreement, **SE**: Standard error, **CI**: 95% Confidence interval, **R**: resistant, **S**: susceptible.

Further comparison between WGS and LPA GenoType MTBDR*sl* results was also undertaken. All strains (140/140) had valid results for both methods and a categorical agreement between 95 to 100% was determined. Kappa values indicated very high degree of agreement for fluoroquinolones (moxifloxacin and levofloxacin) and aminoglycosides (amikacin and kanamycin) and a lesser agreement for capreomycin (k = 0.35). However, this drug presented high positive and negative agreement values (S2 Table).

### Discordant results

Discordant DST genotypic (WGS) and phenotypic (BACTEC MGIT) results were seen on at least one occasion for six compared drugs, except amikacin. 18 of the 140 strains had discordant results between WGS and BACTEC MGIT methods of which seven different strains were categorized as “genotypically susceptible / phenotypically resistant” and 11 were categorized as “genotypically resistant / phenotypically susceptible” strains. 9 of these 11 strains had “associated with resistance” type of mutations whereas one strain had a “associated with resistance–interim” type of mutation, and only one harboured both types of mutations. The nature of discrepancy for each discordant strain are outlined in Table 3. Additionally, for nine different strains there was a discordance between the LPA GenoType MTBDR*sl* result and WGS comparison for fluoroquinolones or capreomycin (and both for one strain). All the mutations detected by WGS are currently catalogued as “associated with resistance–interim” (S3 Table).

**Table 3:** Discordant results obtained for each drug using WGS and BACTEC MGIT.

Drug/Sample	BACTEC	WGS	Mutation	WHO Category
**Rifampicin**				
PER-TB-030	S	R	*rpoB*_Q432L	Assoc w R
PER-TB-092	S	R	*rpoB*_I491F	Assoc w R
**Isoniazid**				
PER-TB-075	R	S	-	
**Pyrazinamide**				
PER-TB-015	S	R	*pncA*_K48T	Assoc w R—Interim
PER-TB-022	R	S	-	
PER-TB-029	R	S	-	
PER-TB-036	R	S	-	
PER-TB-067	R	S	-	
PER-TB-116	S	R	*pncA*_Q10R	Assoc w R
**Capreomycin**				
PER-TB-062	R	S	-	
**Moxifloxacin**				
PER-TB-013	S	R	*gyrA*_A90V	Assoc w R
PER-TB-015	S	R	*gyrA*_D94N	Assoc w R
			*gyrB*_N499D	Assoc w R—Interim
PER-TB-041	S	R	*gyrA*_A90V	Assoc w R
PER-TB-068	S	R	*gyrA*_A90V	Assoc w R
PER-TB-080	S	R	*gyrA*_D94A	Assoc w R
PER-TB-095	S	R	*gyrA*_A90V	Assoc w R
PER-TB-101	S	R	*gyrA*_D94G	Assoc w R
PER-TB-103	S	R	*gyrB*_D461N	Assoc w R—Interim
**Levofloxacin**				
PER-TB-103	S	R	*gyrB*_D461N	Assoc w R—Interim
PER-TB-108	R	S	-	

**WHO**: World Health Organization, **WGS**: Whole genome sequencing, **R**: resistant, **S**: susceptible. **Assoc w R**: Associated with resistance, **Assoc w R–Interim**: Associated with resistance–Interim.

### Genetic relatedness

**Fig 2:**
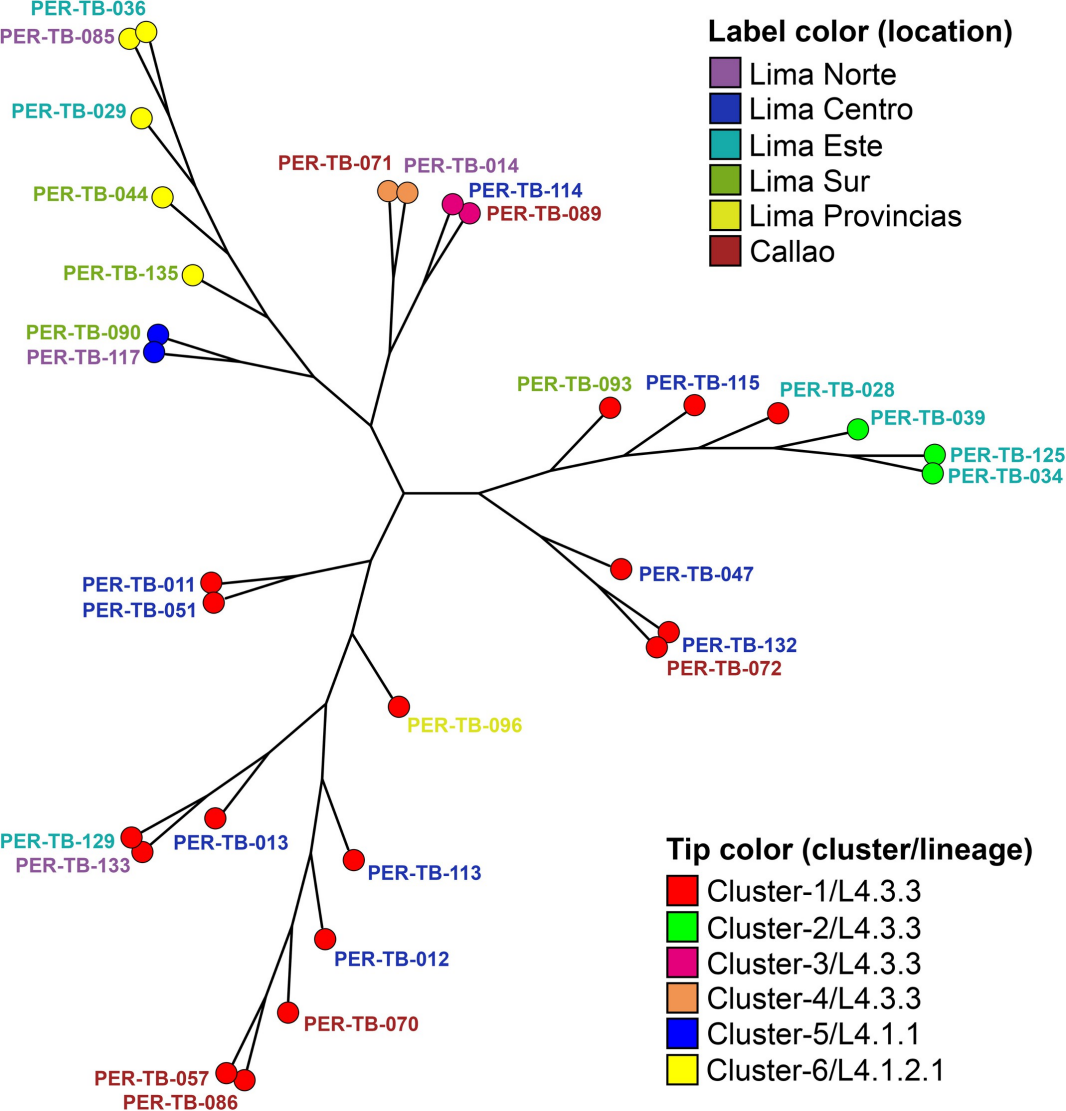
Maximum parsimony tree of samples belonging to clusters of transmission. The tree was generated using six clusters defined by 12 SNP cutoff value. Each tip represents one strain.

In most patients (97.9%,137/140) a single sublineage was identified, of which 134 belonged to “Lineage 4” and three belonged to “Lineage 2”; however, for three samples a mix of strains belonging to two different sublineages were identified. “Lineage 4” strains were constituted of nine different sublineages while within “lineage 2” only the single 2.2.1 sublineage was identified (S4 Table). Using the alternative cutoff values of 5, 10 and 12 SNP distances to define relatedness, the proportion of strains that were clustered were 3.6% (5/140), 17.9% (25/140) and 22.1% respectively. For the 12 SNP threshold six clusters emerged (a total of 31 strains), the largest of which consisted of 17 strains geographically distributed across all parts of Lima and Callao. Sublineage 4.3.3 accounted for 51.4% of all study strains and 77.4% (24/31) of all clustered strains (Fig 2). All strains defined in the same cluster showed the same pattern of drug-resistant mutations for rifampicin, isoniazid, pyrazinamide, ethambutol, and streptomycin, but the same did not happen for moxifloxacin/levofloxacin, capreomycin and ethionamide (S5 Table).

## Discussion

We report for the first time the establishment of WGS into the public health laboratory workflow in Peru, and the first in-country comparison of performance of BACTEC MGIT phenotypic and WGS genotypic DST under routine program conditions. The current health policy in Peru establishes a primary DST for all TB patients using LPA GenoType MTBDR*plus* v2, Xpert MTB/RIF ultra or Microscopic-Observation Drug Susceptibility (MODS) in intermediate or peripheral centers. However, if the primary DST indicates resistance to rifampicin and/or isoniazid, cultures are sent to the LRNM for additional routine testing against second-line agents (including new and repurposed drugs) using the LPA GenoType MTBDR*sl* v2 and BACTEC MGIT methods. Although this approach follows the recommendations of the WHO [21], it is often lengthy and may fail to deliver DST results in a clinically actionable and meaningful timeframe.

Two important drawbacks of phenotypic DST are i) the relatively long culture times (relative to other bacteria) and ii) the risk of contamination or growth failure, which requires a test repetition, increasing the delay for the delivery of results. The problem of the long MTB doubling time is evidenced in the large difference in time to obtain results between BACTEC MGIT and WGS that we, like many previous authors, reported. Although up to 90% of samples from untreated patients may be positive in liquid culture by 14 days, cultures are extended up to 42 days [22]. These timelines vary depending upon the flow and workload in each laboratory, so in our case it took an average of 31 days for the results of the first- and second-line tests to be notified to the patient, and up to 49 days for new and redefined drugs. In contrast our average time to obtain WGS results of 12 days is slightly longer than that obtained by Olaru and colleagues (8 days) [23], but lower than that obtained by Park and colleagues (35 days) [24], both from London (like our calculation the first study did not include primary isolation, though the second did). The reduced time to obtain the complete panel of susceptibility results represents an important advantage of using WGS in laboratory workflows, facilitating DST-guided therapy much earlier. On the other hand, in this study BACTEC MGIT results were ultimately not obtained for 13.6% of samples, although these results were less than half those reported in 2016 [25]. In contrast, the WGS results were obtained for 100% of the samples, representing an important advantage for patients and clinicians.

In general, WGS presented a high diagnostic agreement with routine workflow methods for the compared drugs with exception of moxifloxacin (BACTEC MGIT, k = 0.31) and capreomycin (LPA GenoType MTBDR*sl* v2, k = 0.35). However, the additional analysis of these drugs showed high values of sensitivity (or positive agreement) and specificity (or negative agreement) evidencing the well-known statistical phenomenon called the first kappa paradox caused by symmetrically unbalanced situation of marginal values [26, 27]. For moxifloxacin this phenomenon was enhanced due to the additional detection of eight resistant strains by WGS, identifying in seven of the eight at least one mutation previously characterized as associated with low-level resistance (*gyrA*_A90V, *gyrB*_N499D, *gyrA*_D94A and *gyrB*_D461N) [28]. Therefore, despite the low kappa value and the discordant results, we can be established that WGS is an appropriate tool for the analysis of resistance for both drugs. It was also observed a discordant strain for rifampicin harboring the *rpoB*_I491F mutation, which has previously been characterized as a low-level resistance mutation and associated with relapses [29]. This strain gave a susceptible result by phenotypic testing because it was processed prior to the updated use of the WHO recommended critical concentration of rifampicin 0.5 μg/mL in the LRNM (which occurred near the end of the study) [7]. Previous comparative studies between WGS and routine diagnostic flows also evidenced the presence of discordant results similar to what was obtained in our study, revealing the existence of intrinsic factors of the methods evaluated as the cause of the discordances [30, 31]. Our results reinforce the evidence that WGS can also be used adequately for the detection of resistance against the most important second-line drugs with good performance, beyond initial use only for first-line drugs [32, 33]. The case of new or redefined drugs is different because, due to their recent introduction worldwide, there is still no extensive information on mutations associated with their resistance [5]. This information highlights the capacity of WGS for the complete characterization of genetic alterations associated with different levels of resistance in order to adjust the doses, or exclude the use, of anti-TB drugs.

Our comparative analysis between the LPA GenoType MTBDR*sl* and WGS has highlighted the limitations of current molecular DST technologies (LPA GenoType MTBDR*plus*, LPA GenoType MTBDR*sl*, and Xpert MTB/RIF Ultra) that test a limited number of targets for a limited number of drugs, generating false negative results and facilitating spread of drug resistance in the community [34, 35]. The discordant cases were due to the presence of resistance-associated mutations present in regions not evaluated by the LPA, specifically *tlyA* and *gyrB* genes for resistance to capreomycin and fluoroquinolones, respectively. WGS overcomes this gene coverage limitation by analyzing many genes associated with resistance to the whole range of anti-TB drugs.

Additionally, WGS has been demonstrated to be a high-resolution public health tool for the detection and understanding of transmission groups or outbreaks that epidemiological research or even traditional genotyping methods have failed to detect [17, 36]. For better results, the identification of genomic clusters must work in conjunction with epidemiological research in a representative sampling size. However, the limitations of this study were not having detailed epidemiological information on the patients and having only a small sample size. Despite this, the use of WGS allowed us to detect the existence of close genetic relationships between the strains analyzed. As expected, the genetic clustering information was consistent with the mutations associated with the main anti-TB drugs. Each defined cluster was mostly made up of strains from different areas, evidencing the high flow of people between different points of Lima and Callao. Previous studies in Peru demonstrated the ability of WGS to resolve transmission clusters that were not clearly defined by previous methods or that were not identified in drug-resistant strains [34, 37]. Our results complement these studies, evidencing the important use of WGS to understand in real-time the epidemiology of TB at the national, regional, and local levels.

The costs associated with the development of WGS have decreased considerably making it an attractive option that can compete with current molecular methodologies and comparable to the costs of phenotypic DST with the added advantage of molecular epidemiological data [38]. England was the first country to implement the routine use of WGS and adopt a national network to improve TB diagnosis [39]. Since then, a growing number of countries have established the routine WGS for TB diagnostic and DST [40–43]. WGS was initially implemented in countries with a low TB burden, however, its usefulness in countries with a high TB burden has also been demonstrated [30, 31]. Peru has the highest burden of MDR-TB and Pre-XDR-TB in the American continent [1]. Therefore, the implementation of the routine service of WGS in Peru is a key action in the fight against drug resistance in the region.

In conclusion, the routine use of WGS in laboratory workflows in Peru has a high diagnostic accuracy to detect resistance against key current anti-TB drugs, allowing results to be obtained through a single analysis and quickly cutting the chain of transmission of drug-resistant TB in the Peruvian community.

## Supporting information

S1 Fig Workflow designed for the study. The figure shows the flow of strains sent to the LRNM for routine drug DST using the genotypic LPA GenoType MTBDR*sl* and the phenotypic BACTEC MGIT methods, alongside the parallel work carried out using WGS for the study.
(PDF)

S2 Fig Geographic origin of all study samples. Place of origin of the samples analysed in the study. The map shows the entire extension of the department of Lima (capital of Peru). The existing limits between the districts of Lima and Callao are delineated. Numerical values specify the amounts of samples analysed in each district. Map was generated using QGIS v3.14.15.
(PDF)

S1 Table Turnaround times for results by routine and WGS workflows. Times are measured from assignation of samples to LRNM’s routine workflow until the date in which the BACTEC MGIT results are issued to the patient or the WGS results are obtained and validated by the system.
(PDF)

S2 Table Agreement results between WGS and LPA GenoType MTBDR*sl*. Categorical and statistical agreements between the DST results of the WGS and the LPA GenoType MTBDR*sl* were calculated for second-line anti-TB drugs.
(PDF)

S3 Table Discrepant results between WGS and LPA GenoType MTBDR*sl*. In-depth analysis of DST discordant results between WGS and LPA GenoType MTBDR*sl* for the fluoroquinolones (moxifloxacin and levofloxacin) and capreomycin drugs. The mutations detected by WGS are specified for each strain. Likewise, the annotation of the category of resistance association determined by the WHO mutation catalogue of 2021 is assigned. BACTEC results are added for comparison.
(PDF)

S4 Table Lineage distribution of Peruvian MTB strains. Lineages and sublineages determined in the MTB strains included in the study from Lima and Callao (MDR-TB *hot-spot* areas in Peru).
(PDF)

S5 Table Genetic characteristics of MTB samples belonging to transmission clusters. 2, 5 and 6 clusters were obtained using 5, 10 and 12 SNPs cut-off values, respectively. The color graduation denotes the number of clusters, and the strains that are members of each one, that were obtained using the different cutoff values (white boxes do not denote any cluster).
(PDF)
