# Supplementary material for: Whole Genome Sequencing of Mycobacterium tuberculosis under routine conditions in a high-burden area of multidrug-resistant tuberculosis in Peru
Source: PLoS One. 2024 Jun 11;19(6):e0304130. doi: 10.1371/journal.pone.0304130 (PMC11166294; doi:10.1371/journal.pone.0304130)
Supplement: S1 Fig — The figure shows the flow of strains sent to the LRNM for routine drug DST using the genotypic LPA GenoType MTBDRsl and the phenotypic BACTEC MGIT methods, alongside the parallel work carried out using WGS for the study. (PDF) [file pone.0304130.s001.pdf]

S1 Fig: Workflow designed for the study.

The figure shows the flow of strains sent to the LRNM for routine DST using the genotypic LPA GenoType MTBDR*sl* and the phenotypic BACTEC MGIT methods, alongside the parallel work carried out using WGS for the study.

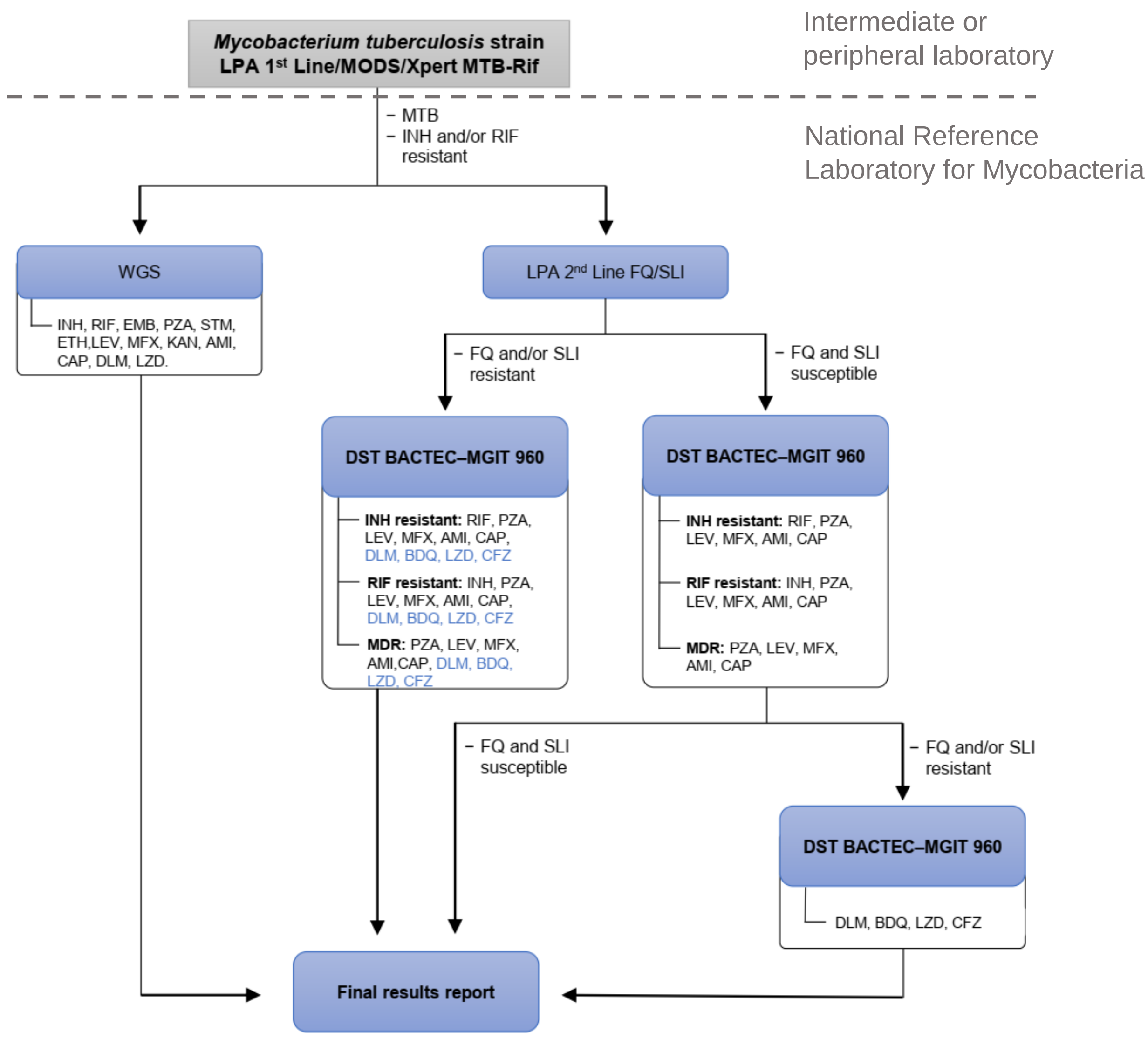

DST: Drug Susceptibility Test, **MGIT**: Mycobacterial Growth Indicator Tube, **WGS**: Whole Genome Sequencing, **LPA**: Line probe Assay, **FQ**: Fluoroquinolone, **SLI**: Second-Line Injectable, **MODS**: Microscopic-Observation Drug Susceptibility assay, **INH**: isoniazid, **RIF**: rifampicin, **EMB**: ethambutol, **PZA**: pyrazinamide, **STM**: streptomycin, **ETH**: ethionamide, **LEV**: levofloxacin, **MFX**: moxifloxacin, **KAN**: kanamycin, **AMI**: amikacin, **CAP**: capreomycin, **DLM**: delamanid, **BDQ**: bedaquiline, **LZD**: linezolid, **CFZ**: clofazimine.
