# Supplementary material for: Whole Genome Sequencing of Mycobacterium tuberculosis under routine conditions in a high-burden area of multidrug-resistant tuberculosis in Peru
Source: PLoS One. 2024 Jun 11;19(6):e0304130. doi: 10.1371/journal.pone.0304130 (PMC11166294; doi:10.1371/journal.pone.0304130)

S2 Fig: Geographic origin of all study samples.

Place of origin of the samples analysed in the study. The map shows the entire extension of the department of Lima (capital of Peru). The existing limits between the districts of Lima and Callao are delineated. Numerical values specify the amounts of samples analysed in each district. Map was generated using QGIS v3.14.15.

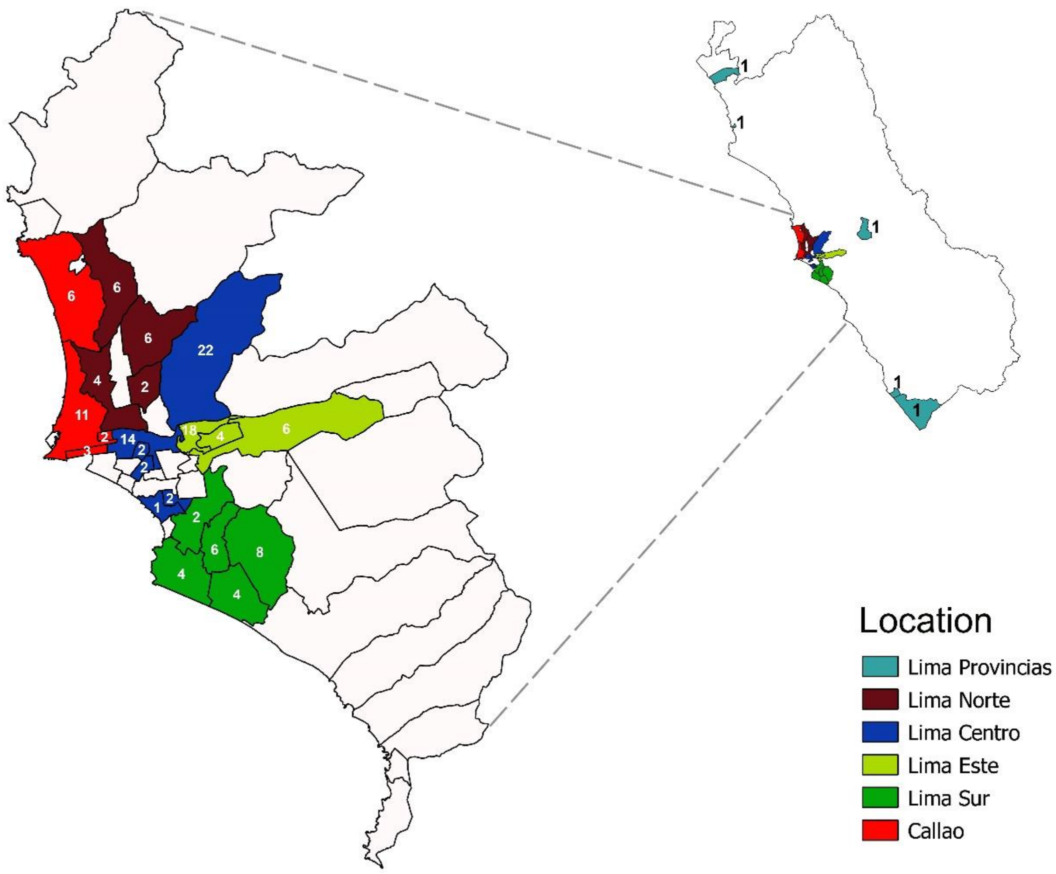

Supplement: S2 Fig — Place of origin of the samples analysed in the study. The map shows the entire extension of the department of Lima (capital of Peru). The existing limits between the districts of Lima and Callao are delineated. Numerical values specify the amounts of samples analysed in each district. Map was generated using QGIS v3.14.15. (PDF) [file pone.0304130.s002.pdf]
