# Supplementary material for: Whole Genome Sequencing of Mycobacterium tuberculosis under routine conditions in a high-burden area of multidrug-resistant tuberculosis in Peru
Source: PLoS One. 2024 Jun 11;19(6):e0304130. doi: 10.1371/journal.pone.0304130 (PMC11166294; doi:10.1371/journal.pone.0304130)
Supplement: S1 Table — Times are measured from assignation of samples to LRNM’s routine workflow until the date in which the BACTEC MGIT results are issued to the patient or the WGS results are obtained and validated by the system. (PDF) [file pone.0304130.s003.pdf]

S1 Table: Turnaround times for results by routine and WGS workflows.

Times are measured from assignation of samples to the LRNM's routine workflow until the date in which the BACTEC MGIT results are issued to the patient or the WGS results are obtained and validated by the system.

**Method**	**Drug**	**Times (days)**			
		**Minimum**	**Maximum**	**Mean (SD)**	**Median (IQR)**
BACTEC	INH	22.4	71.7	35.5 (15.3)	30.5 (25 – 34)
	RIF	14.7	63.7	29.6 (7.4)	28.7 (23 – 34)
	PZA	14.7	71.7	30.2 (8.8)	28.7 (24 – 33)
	AMI	14.7	71.7	29.0 (6.8)	28.6 (24 – 33)
	CAP	14.7	71.7	29.3 (7.7)	28.6 (24 – 33)
	MFX	14.7	71.7	29.0 (6.8)	28.6 (24 – 33)
	LEV	14.7	71.7	29.2 (7.1)	28.6 (24 – 33)
	BDQ	27.7	118.7	53.2 (26.3)	52.6 (28 – 69)
	LZD	26.7	118.7	48.9 (25.9)	43.6 (28 – 61)
	CFZ	26.7	118.7	46.8 (26.4)	31.7 (28 – 60)
	DLM	26.7	118.7	46.8 (26.4)	31.7 (28 – 60)
WGS	All drugs*	9.5	18.5	12.0 (2.2)	11.5 (9 – 13)

WGS: Whole Genome sequencing, **SD**: standard deviation, **IQR**: Interquartile range, **INH**: isoniazid, **RIF**: rifampicin, **PZA**: pyrazinamide, **LEV**: levofloxacin, **MFX**: moxifloxacin, **AMI**: amikacin, **CAP**: capreomycin, **DLM**: delamanid, **BDQ**: bedaquiline, **LZD**: linezolid, **CFZ**: clofazimine. ***13** drugs evaluated by the 2021 WHO catalogue.
