# Supplementary material for: Whole Genome Sequencing of Mycobacterium tuberculosis under routine conditions in a high-burden area of multidrug-resistant tuberculosis in Peru
Source: PLoS One. 2024 Jun 11;19(6):e0304130. doi: 10.1371/journal.pone.0304130 (PMC11166294; doi:10.1371/journal.pone.0304130)
Supplement: S2 Table — Categorical and statistical agreements between the DST results of the WGS and the LPA GenoType MTBDRsl were calculated for second-line anti-TB drugs. (PDF) [file pone.0304130.s004.pdf]

S2 Table: Agreement results between WGS and LPA GenoType MTBDR*sl*.

Categorical and statistical agreements between the DST results of the WGS and the LPA GenoType MTBDR*sl* were calculated for second-line anti-TB drugs.

Drug	Categorical agreement (%)	Positive agreement	Negative agreement	Kappa	
				value (CI)	SE
MFX/LFX	97.9	0.90	0.98	0.85 (0.67 - 1.00)	0.087
AMI/KAN	100.0	1.00	1.00	1.00 (1.00 - 1.00)	0.000
CAP	95.0	1.00	0.95	0.35 (-0.01 - 0.71)	0.182

MFX: Moxifloxacin, **LFX**: Levofloxacin, **AMI**: Amikacin, **KAN**: Kanamycin, **CAP**: Capreomycin, **CI**: 95% Confidence interval, **SE**: Standard error.
