# Supplementary material for: Whole Genome Sequencing of Mycobacterium tuberculosis under routine conditions in a high-burden area of multidrug-resistant tuberculosis in Peru
Source: PLoS One. 2024 Jun 11;19(6):e0304130. doi: 10.1371/journal.pone.0304130 (PMC11166294; doi:10.1371/journal.pone.0304130)
Supplement: S3 Table — In-depth analysis of DST discordant results between WGS and LPA GenoType MTBDRsl for the fluoroquinolones (moxifloxacin and levofloxacin) and capreomycin drugs. The mutations detected by WGS are specified for each strain. Likewise, the annotation of the category of resistance association determined by the WHO mutation catalogue of 2021 is assigned. BACTEC results are added for comparison. (PDF) [file pone.0304130.s005.pdf]

S3 Table: Discrepant results between WGS and LPA GenoType MTBDR*sl*.

In-depth analysis of DST discordant results between WGS and LPA GenoType MTBDR*sl* for the fluoroquinolones (moxifloxacin and levofloxacin) and capreomycin drugs. The mutations detected by WGS are specified for each strain. Likewise, the annotation of the category of resistance association determined by the WHO mutation catalogue of 2021 is assigned. BACTEC results are added for comparison.

Drug	LPA	WGS	BACTEC	Mutation	WHO Category
Moxifloxacin/Levofloxacin					
PER-TB-012	R	S	S	-	
PER-TB-103	S	R	S	*gyrB*_D461N	Assoc w R - Interim
PER-TB-107	S	R	NA	*gyrB*_D461N	Assoc w R - Interim
Capreomycin					
PER-TB-005	S	R	R	*tlyA*_G232D	Assoc w R - Interim
PER-TB-012	S	R	R	*tlyA*_G232D	Assoc w R - Interim
PER-TB-057	S	R	R	*tlyA*_G232D	Assoc w R - Interim
PER-TB-070	S	R	R	*tlyA*_G232D	Assoc w R - Interim
PER-TB-086	S	R	R	*tlyA*_G232D	Assoc w R - Interim
PER-TB-105	S	R	NA	*tlyA*_G232D	Assoc w R - Interim
PER-TB-129	S	R	R	*tlyA*_G232D	Assoc w R - Interim

LPA: Line probe assay, **WGS**: Whole genome sequencing, **WHO**: World health organization, **Assoc w R – Interim**: Associated with resistance – Interim. **S**: sensible, **R**: resistant, **NA**: not available.
