# Supplementary material for: Whole Genome Sequencing of Mycobacterium tuberculosis under routine conditions in a high-burden area of multidrug-resistant tuberculosis in Peru
Source: PLoS One. 2024 Jun 11;19(6):e0304130. doi: 10.1371/journal.pone.0304130 (PMC11166294; doi:10.1371/journal.pone.0304130)
Supplement: S4 Table — Lineages and sublineages determined in the MTB strains included in the study from Lima and Callao (MDR-TB hot-spot areas in Peru). (PDF) [file pone.0304130.s006.pdf]

S4 Table: Lineage distribution of Peruvian MTB strains.

Lineages and sublineages determined in the MTB strains included in the study from Lima and Callao (MDR-TB hot-spot areas in Peru).

Lineage	Sublineage	N	%
Lineage 2 (East-Asian)	L2.2.1	3	2.1
Lineage 4 (Euro-American)	L4	9	6.4
	L4.1.1	5	3.6
	L4.1.1.3	4	2.9
	L4.1.2.1	23	16.4
	L4.3.2	3	2.1
	L4.3.3	69	49.3
	L4.3.4.1	3	2.1
	L4.3.4.2	6	4.3
	L4.8	11	7.9
	L4.9	1	0.7
Mixed strains	L4.3.3; L2.2.1	1	0.7
	L4.3.3; L4.1.1	1	0.7
	L4.3.3; L4.1.2.1	1	0.7
**Total**		140	100
