# Supplementary material for: Whole Genome Sequencing of Mycobacterium tuberculosis under routine conditions in a high-burden area of multidrug-resistant tuberculosis in Peru
Source: PLoS One. 2024 Jun 11;19(6):e0304130. doi: 10.1371/journal.pone.0304130 (PMC11166294; doi:10.1371/journal.pone.0304130)
Supplement: S5 Table — 2, 5 and 6 clusters were obtained using 5, 10 and 12 SNPs cut-off values, respectively. The color graduation denotes the number of clusters, and the strains that are members of each one, that were obtained using the different cutoff values (white boxes do not denote any cluster). (PDF) [file pone.0304130.s007.pdf]

S5 Table: Genetic characteristics of MTB samples belonging to transmission clusters.

2, 5 and 6 clusters were obtained using 5, 10 and 12 SNPs cut-off values, respectively. The colour graduation denotes the number of clusters, and the strains that are members of each one, that were obtained using the different cutoff values (white boxes do not denote any cluster).

Sample	Cutoff 5 SNP	10 SNP	12 SNP	Lineage	Location	Drug-resistant type	rifampicin	isoniazid	pyrazinamide	ethambutol	streptomycin	moxifloxacin levofloxacin	capreomycin	ethionamide
PER-TB-013				4.3.3	Lima Centro	Pre-XDR-TB	rpoB_D435V	katG_S315T	pncA_Q10R	embB_Y319S	gid_P84L	gyrA_A90V	-	-
PER-TB-133				4.3.3	Lima Norte	MDR-TB	rpoB_D435V	katG_S315T	pncA_Q10R	embB_Y319S	gid_P84L	-	-	-
PER-TB-057				4.3.3	Callao	MDR-TB	rpoB_D435V	katG_S315T	pncA_Q10R	embB_Y319S	gid_P84L	-	tlyA_G232D	-
PER-TB-070				4.3.3	Callao	MDR-TB	rpoB_D435V	katG_S315T	pncA_Q10R	embB_Y319S	gid_P84L	-	tlyA_G232D	-
PER-TB-086				4.3.3	Callao	MDR-TB	rpoB_D435V	katG_S315T	pncA_Q10R	embB_Y319S	gid_P84L	-	tlyA_G232D	-
PER-TB-011				4.3.3	Lima Centro	MDR-TB	rpoB_D435V	katG_S315T	pncA_Q10R	embB_Y319S	gid_P84L	-	-	ethA_c.752dupG
PER-TB-012				4.3.3	Lima Centro	MDR-TB	rpoB_D435V	katG_S315T	pncA_Q10R	embB_Y319S	gid_P84L	-	tlyA_G232D	-
PER-TB-047				4.3.3	Lima Centro	MDR-TB	rpoB_D435V	katG_S315T	pncA_Q10R	embB_Y319S	gid_P84L	-	-	-
PER-TB-051				4.3.3	Lima Centro	MDR-TB	rpoB_D435V	katG_S315T	pncA_Q10R	embB_Y319S	gid_P84L	-	-	-
PER-TB-028				4.3.3	Lima Este	MDR-TB	rpoB_D435V	katG_S315T	pncA_Q10R	embB_Y319S	gid_P84L	-	-	-
PER-TB-072				4.3.3	Callao	MDR-TB	rpoB_D435V	katG_S315T	pncA_Q10R	embB_Y319S	gid_P84L	-	-	-
PER-TB-093				4.3.3	Lima Sur	MDR-TB	rpoB_D435V	katG_S315T	pncA_Q10R	embB_Y319S	gid_P84L	-	-	-
PER-TB-096				4.3.3	Lima Provincias	MDR-TB	rpoB_D435V	katG_S315T	pncA_Q10R	embB_Y319S	gid_P84L	-	-	-
PER-TB-113				4.3.3	Lima Centro	MDR-TB	rpoB_D435V	katG_S315T	pncA_Q10R	embB_Y319S	gid_P84L	-	-	ethA_c.1431dupT
PER-TB-129				4.3.3	Lima Este	MDR-TB	rpoB_D435V	katG_S315T	pncA_Q10R	embB_Y319S	gid_P84L	-	tlyA_G232D	-
PER-TB-132				4.3.3	Lima Centro	MDR-TB	rpoB_D435V	katG_S315T	pncA_Q10R	embB_Y319S	gid_P84L	-	-	-
PER-TB-115				4.3.3	Lima Centro	MDR-TB	rpoB_D435V	katG_S315T	pncA_Q10R	embB_Y319S	gid_P84L	-	-	-
PER-TB-125				4.3.3	Lima Este	MDR-TB	rpoB_D435V	katG_S315T	pncA_Q10R	embB_Y319S	-	-	-	ethA_c.752dupG
PER-TB-034				4.3.3	Lima Este	MDR-TB	rpoB_D435V	katG_S315T	pncA_Q10R	embB_Y319S	-	-	-	-
PER-TB-039				4.3.3	Lima Este	MDR-TB	rpoB_D435V	katG_S315T	pncA_Q10R	embB_Y319S	-	-	-	-
PER-TB-089				4.3.3	Callao	MDR-TB	rpoB_S450L	inhA_c-15t	pncA_H51R	-	-	-	-	inhA_c-15t
PER-TB-114				4.3.3	Lima Centro	MDR-TB	rpoB_S450L	inhA_c-15t	pncA_H51R	-	-	-	-	inhA_c-15t
PER-TB-071				4.3.3	Callao	MDR-TB	rpoB_S450L	inhA_c-15t	pncA_H51R	embB_M306I	-	-	-	inhA_c-15t
PER-TB-014				4.3.3	Lima Norte	MDR-TB	rpoB_S450L	inhA_c-15t	pncA_H51R	embB_M306I	-	-	-	inhA_c-15t
PER-TB-090				4.1.1	Lima Sur	MDR-TB	rpoB_S450L	katG_S315T	pncA_Q10P	embB_D354A	-	-	-	-
PER-TB-117				4.1.1	Lima Norte	MDR-TB	rpoB_S450L	katG_S315T	pncA_Q10P	embB_D354A	-	-	-	-
PER-TB-029				4.1.2.1	Lima Este	HR-TB	-	katG_S315T, inhA_c-15t	-	-	gid_G73A	-	-	inhA_c-15t, ethA_S399*
PER-TB-044				4.1.2.1	Lima Sur	HR-TB	-	katG_S315T, inhA_c-15t	-	-	gid_G73A	-	-	inhA_c-15t, ethA_S399*
PER-TB-036				4.1.2.1	Lima Este	HR-TB	-	katG_S315T, inhA_c-15t	-	-	gid_G73A	-	-	inhA_c-15t, ethA_S399*
PER-TB-135				4.1.2.1	Lima Sur	HR-TB	-	katG_S315T, inhA_c-15t	-	-	gid_G73A	-	-	inhA_c-15t, ethA_S399*
PER-TB-085				4.1.2.1	Lima Norte	HR-TB	-	katG_S315T, inhA_c-15t	-	-	gid_G73A	-	-	inhA_c-15t, ethA_S399*

TB: tuberculosis, **HR-TB**: Mono-resistant to isoniazid TB, **MDR-TB**: multidrug-resistant TB, **Pre-XDR-TB**: Pre extensively resistant TB. ***** = translation termination codon (*stop codon*).
